# Iron imbalance in cancer: Intersection of deficiency and overload

**DOI:** 10.1002/cam4.4761

**Published:** 2022-04-22

**Authors:** Tulika Basak, Rupinder Kaur Kanwar

**Affiliations:** ^1^ Institute for Innovation in Mental and Physical Health and Clinical Translation (IMPACT) School of Medicine, Faculty of Health, Deakin University Geelong Victoria Australia; ^2^ Department of Translational Medicine Centre All India Institute of Medical Sciences (AIIMS) Bhopal Bhopal India

**Keywords:** anaemia, cancer, inflammation, iron, iron deficiency, iron overload, reactive oxygen species (ROS)

## Abstract

Iron, an essential trace element, plays a complex role in tumour biology. While iron causes cancer clearance through toxic free radical generation, iron‐induced free radical flux also acts as a cancer promoter. These fates majorly guided through cellular response towards pro‐oxidant and antioxidant settings in a tumour microenvironment, designate iron‐induced oxidative stress as a common yet paradoxical factor in pro‐tumorigenesis as well as anti‐tumorigenesis, posing a challenge to laying down iron thresholds favouring tumour clearance. Additionally, complexity of iron's association with carcinogenesis has been extended to iron‐induced ROS's involvement in states of both iron deficiency and overload, conditions identified as comparable, inevitable and significant coexisting contributors as well as outcomes in chronic infections and tumorigenesis. Besides, iron overload may also develop as an unwanted outcome in certain cancer patients, as a result of symptomatic anaemia treatment owed to irrational iron‐restoration therapies without a prior knowledge of body's iron status with both conditions synergistically acting towards tumour aggravation. The co‐play of iron deficiency and overload along with iron's pro‐tumour and antitumour roles with intersecting mechanisms, thus presents an unpredictable regulatory response loop in a state of malignancy. The relevance of iron's thresholds beyond which it proves to be beneficial against tumorigenesis hence becomes questionable. These factors pose a challenge, over establishing if iron chelation or iron flooding acts as a better approach towards antitumour therapies. This review presents a critical picture of multiple contrasting features of iron's behaviour in cancer, leading towards two conditions lying at opposite ends of a spectrum: iron deficiency and overload in chronic disease conditions including cancer, hence, validating the critical significance of diagnosis of patients' iron status prior to opting for subsequent therapies.

## Graphical abstract

For majority of physicians, the primary concern in terms of “iron” till date, remains anaemia or simply a state of iron deficiency, without comprehending ‘iron overload’ as a more prevalent and leading issue, and substantially more life‐threatening, leading to a myriad of complications. Cancer being a dynamic disease may provoke co‐existent states of iron‐deficiency and overload at varied time points in a single patient. Unmethodical red cell transfusions and iron supplementations established as default treatment strategies for iron deficiency may open a ‘Pandora’s box’, promoting unwanted risks of iron overload, a pre‐cursor to tumorigenesis and a condition that is often overlooked.

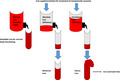

## 1 INTRODUCTION

Iron, an essential trace element, participates as a cofactor in several enzymes required in biochemical functions in human physiology.^1^ Its behaviour is attributed to its potential to readily donate and accept electrons, thus inter‐switching between insoluble ferric (Fe^+3^) and soluble ferrous (Fe^+2^) forms.^1^ However, this redox ability makes iron a paradoxical element, due to its potential toxicity towards cells under imbalanced conditions, despite being a key factor in normal anatomical functioning.^2^

Iron imbalance in the body occurs when regulatory processes for intracellular iron maintenance function abnormally, or iron intake exceeds the required physiological range.^2^ Both conditions of iron deficiency and overload may be detrimental to optimal body functioning, consequentially catalysing cancer incidence, recurrence and chronic infections.^3^ Additionally, with iron chelation as an approach towards anticancer therapy, iron‐driven oxidative injury presents a contrasting view on the role of iron in anticancer applications.^4^ Besides, irrational iron administration or chronic red blood cell (RBC) transfusion therapies clinically prescribed for treatment of iron deficient or chemotherapy‐induced anaemia (CIA), may lead to unwanted risks of iron overload, further aggravating the disease.^5^ One such example is cancer‐driven chronic anaemia clinically indicated by deficient available or functional iron that might render iron administration counterproductive, thus intensifying risks of malignancy.^5^ In this review, we, therefore, discuss a possible link on the contradictory role of iron deficiency and overload in chronic conditions including cancer, signifying the diagnosis of iron status in cancer patients prior to opting for iron‐restoration strategies.

## 2 IRON‐REGULATORY MECHANISM

**FIGURE 1:**
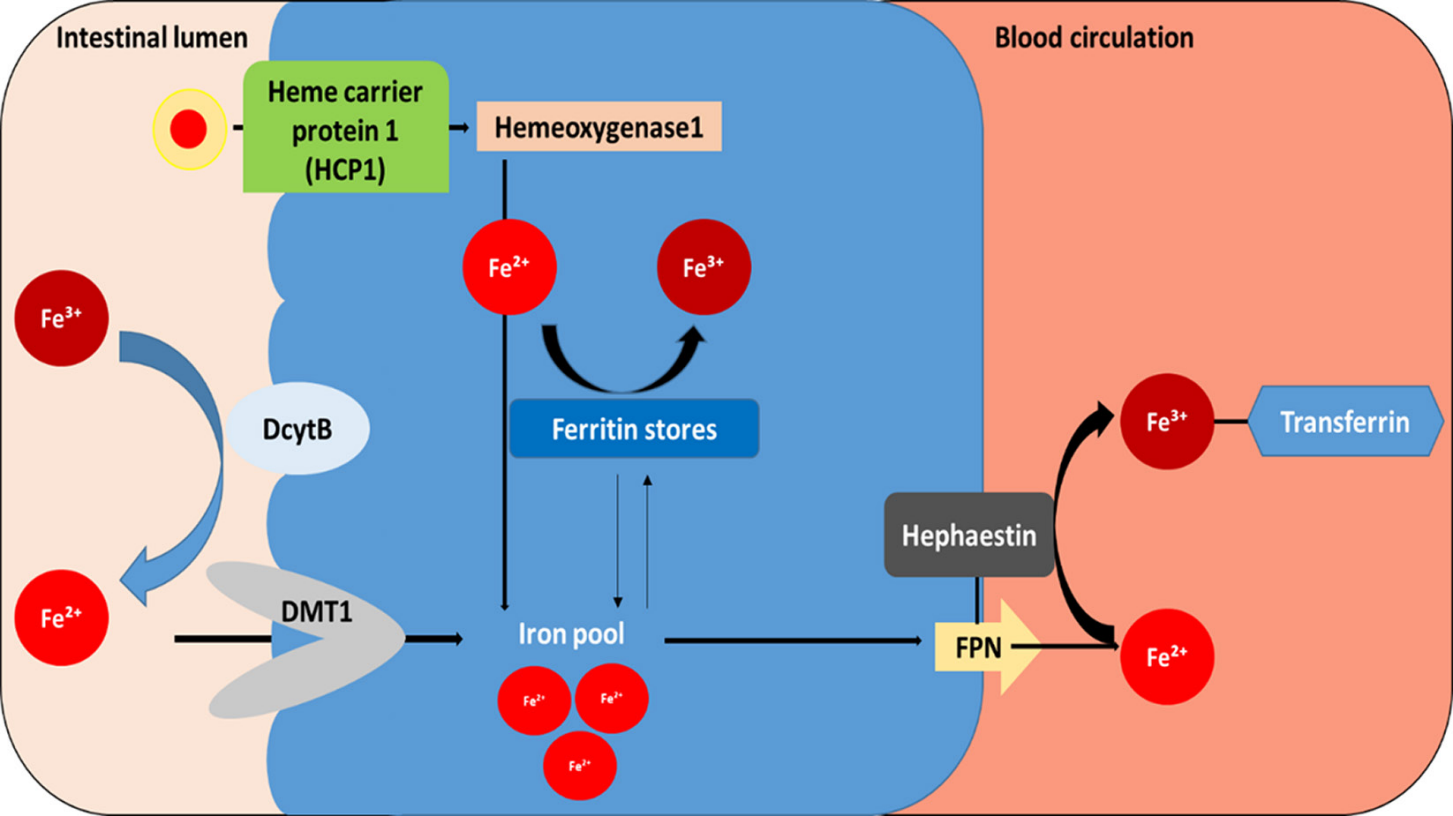
A schematic representation of iron‐regulatory axis––from intestinal lumen to circulation. Reduced iron is absorbed by duodenal enterocytes via divalent metal transporter 1 (DMT1) or heme carrier protein‐1 (HCP‐1), then transported to blood vessels via ferroportin (FPN), the major iron exporter. Hepcidin‐FPN interplay in liaison with a transmembrane ferroxidase, hephaestin controls intestinal iron absorption, plasma iron concentration and its circulatory release throughout the body. Ferric ion (Fe^+3^) complexed with transferrin (Tf) is further delivered to bone marrow which gets transported to peripheral tissues through receptor‐mediated delivery, for essential functions. Excessive iron is stored as ferritin reserves until required for erythropoiesis. *Abbreviation*: DcytB = Duodenal cytochrome B.

A healthy human body contains approximately 3–4 grams of iron, majorly intracellularly available, with an average of 64% circulating as haemoglobin (Hb) in erythrocytes, or as stored as ferritin reserves in hepatocytes, along with about 21% contained in macrophages, and 14% in myoglobin and iron‐containing enzymes in various cell types.^6^, ^7^ Blood plasma, on the other hand, contains minor amounts of iron in the range of a few milligrams with nearly all of it bound to iron‐binding plasma glycoprotein, transferrin (Tf), with its iron‐binding sites approximately 20%–40% saturated with ferric (Fe^+3^) atoms.^6^ Under optimal health conditions, dietary or cellular iron in Fe^+3^ form complexed with Tf binds to the cell surface transferrin receptor (TfR), further internalised through endocytosis via receptor‐mediated delivery.^8^ Fe^+3^ is then released from the complex due to the acidic environment of the early endosome, in order to undergo reduction to ferrous form by an enterocytic ferrireductase, duodenal cytochrome B (DcytB).^8^, ^9^ However, with DcytB reported to exhibit low expression in erythroid precursor cells, an NADPH dependent, membrane bound erythroid protein, the six‐transmembrane epithelial antigen of prostate 3 (STEAP3) was identified as yet another ferrireductase responsible for endosomal iron reduction.^9^, ^10^ Post reduction, Fe^+2^ is delivered to the cytoplasm by duodenal enterocytes via divalent metal transporter 1 (DMT1), known to be selective for Fe^+2^, post which Fe^+2^ is released into circulation via ferroportin (FPN), the major iron exporter, rendering it available for oxidation by a transmembrane ferroxidase, hephaestin.^8^, ^9^, ^10^ Figure 1 schematically represents the general mechanism of cellular iron metabolism in a healthy human body. Resulting ferric ions complexed with Tf are further delivered to bone marrow which get transported to peripheral tissues through receptor‐mediated delivery, for essential functions.^8^ Excessive iron is stored as ferritin reserves in macrophages, until required for erythropoiesis.^8^ Erythropoietin being the RBC regulator, requires the release of stored iron via FPN for Hb synthesis.^11^ FPN regulation is partially driven by hepcidin, a hepatic peptide identified as the systemic iron regulator.^11^ Hepcidin‐FPN interplay in liaison with, hephaestin, therefore, controls intestinal iron absorption, plasma iron concentration and its circulatory release throughout the body.^12^

**FIGURE 2:**
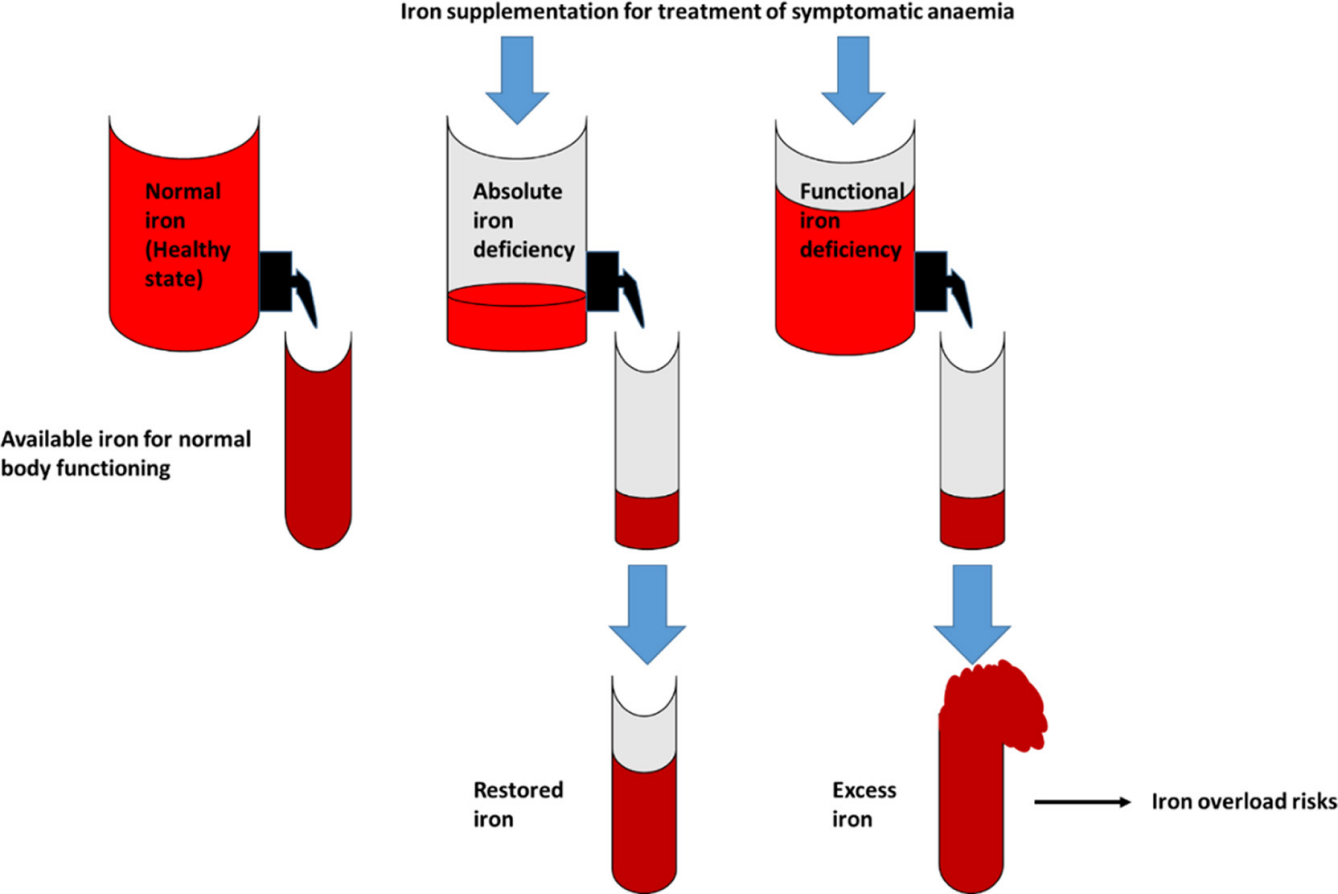
A diagrammatic representation of states of absolute and functional iron deficiencies (AID and FID, respectively) as compared to healthy iron status. FID and AID are characterised by significant reduction or unavailability of iron for erythropoiesis, thereby resulting in clinical anaemia in chronic inflammations, including cancer. FID is a condition of iron deficiency marked by adequate or increased iron stores with elevated hepcidin, while this iron is inaccessible for erythropoiesis. AID, on the other hand, indicates a complete depletion of iron reserves and iron unavailability for red blood cell (RBC) synthesis.

The iron‐regulatory mechanism is however disrupted in cancer and cancer‐associated chronic inflammation. Tumour microenvironment is actively regulated and modulated by inflammatory cells through a single or a concerted effect of several cytokines including interleukins, oncostatin and leptins.^13^, ^14^ This induces hepcidin release, thus disrupting the FPN/hepcidin channel, and inhibiting macrophageal iron export, leading to an ‘iron‐blockage’.^13^, ^14^ Resulting reduced serum iron and increased ferritin stores lead to a state of ‘functional iron deficiency’ (FID) in cancer patients, one of the predominant mechanisms in cancer‐associated iron deficit.^15^ FID as a response to cancer is critical, since it is noteworthy that while cancer patients possess adequate or increased iron stores with elevated hepcidin, this iron is inaccessible for erythropoiesis.^15^ FID prevalence increases with cancer stages and is correlated to treatment‐associated underperformance in cancer patients.^15^ Alternately, iron deficiency may also be absolute abbreviated as AID, marked by a complete depletion of iron reserves and iron unavailability for RBC synthesis.^15^ Differences between FID and AID have been illustrated in Figure 2. Taken together, the common event in both AID and FID is a significant reduction or unavailability of iron for erythropoiesis, thereby resulting in clinical anaemia in chronic inflammations, including cancer.

Interestingly, while anaemia of chronic diseases (ACD) indicates a state of iron deficiency, it has been reflected to associate with the phenotypic expression of hereditary hemochromatosis (HH), an iron overload disorder.^16^ Unsurprisingly, inflammation‐induced expression of HFE, a distinguished contributor in HH has been previously hypothesised to associate with ACD acting through iron blockage in reticuloendothelial cells via inhibition of FPN‐mediated iron export, therefore, implying a possible link between states of iron deficiency and overload.^17^, ^18^ Mutations in iron‐regulatory genes, including *HFE* result in functional hepcidin deficiency and duodenal FPN overexpression, thus causing iron over‐absorption in the gastrointestinal tract, one of the suspected contributors in malignancy, particularly hepatic and colorectal cancers.^19^ Resulting increased intestinal iron transfer to plasma saturates Tf, releasing free iron known as ‘non‐transferrin bound iron’ (NTBI).^20^ NTBI is potent reactive oxygen species (ROS) generator resulting in tissue injury and lipid peroxidation, eventually inducing DNA damage and therefore, are drivers for cancer progression.^21^ This ability is exploited by hepatocytes, predominantly parenchymal cells, being sites for FPN expression and having limited iron export ability, resulting in excessive hepatocyte iron flush due to hepcidin deficiency.^22^ Paradoxically, low hepcidin production as a result of iron deficiency has also been associated with risks of chronic liver complications and hepatic cancer.^23^, ^24^

The above‐mentioned factors cumulatively indicate a possible association between iron deficiency and overload in cancer that might be owed to uncondoned iron‐restoration therapies, an underappreciated topic over the years, and therefore, needs to be individually discussed.

## 3 IRON IMBALANCE AND ROSLINK IN CANCER

**FIGURE 3:**
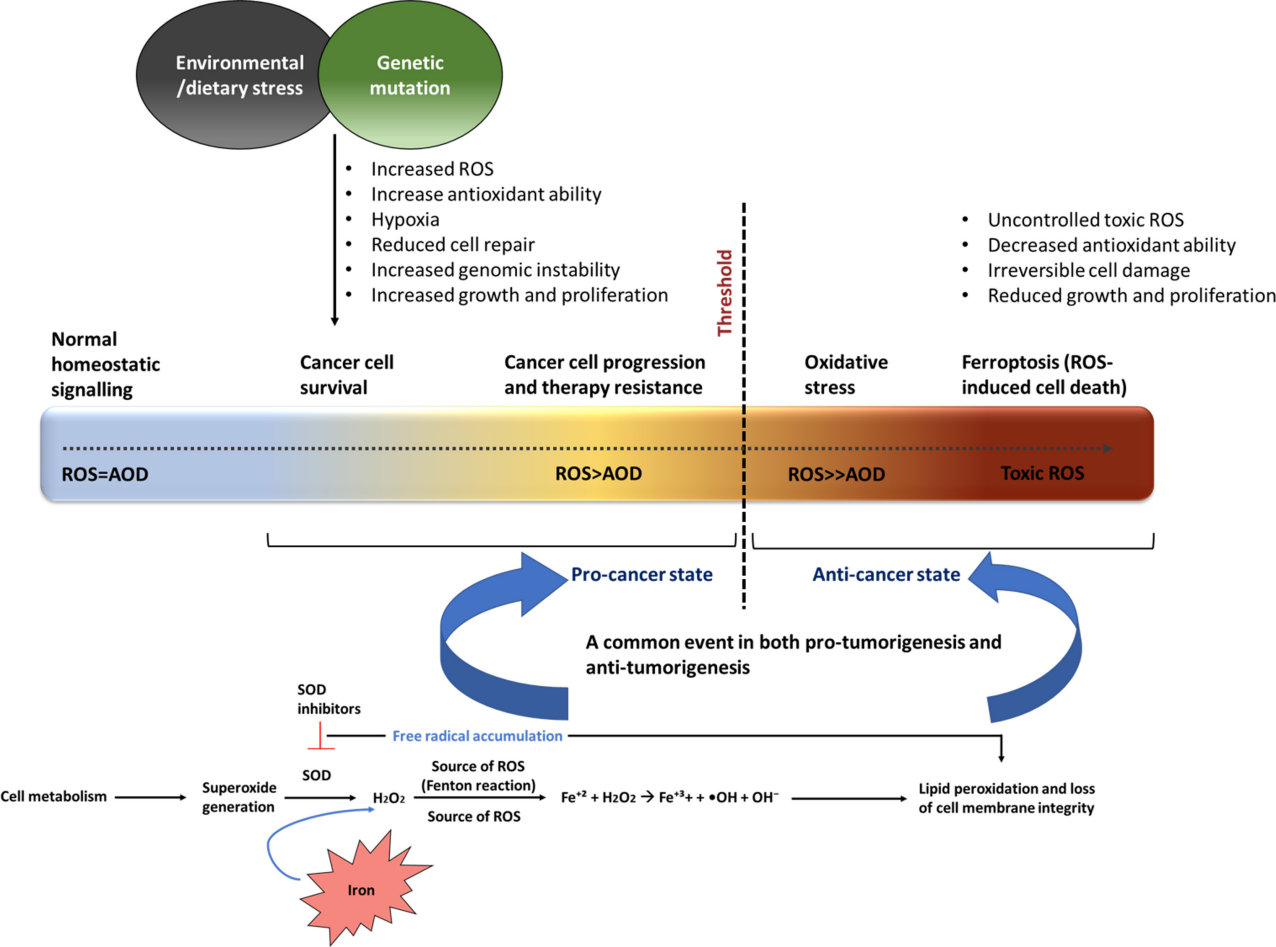
A schematic representation of the dual role of reactive oxygen species (ROS) in cancer. Oxidative stress in a tumour microenvironment promotes superoxide dismutase (SOD) catalysed break down of superoxides into hydrogen peroxide, which in presence of excess free undergoes Fenton reaction leading to an overaccumulation of free radicals/ROS, responsible for lipid peroxidation and loss of cell membrane integrity, a common event favouring both pro‐tumorigenesis and anti‐tumorigenesis, the fate being majorly dependent on comparative ROS levels against cellular antioxidant defence (AOD). While iron‐generated free radicals may stimulate anti‐tumorigenic signalling through ferroptosis induction and drive oxidative stress‐promoted tumour clearance, cancer cells possess the ability to adapt to the hypoxic environment, triggering cellular transformation pathways through irreversible cell membrane damage, therefore, facilitating their survival and proliferation.

A major factor behind the iron‐cancer link is iron's ability to undergo redox reactions, the source of hypoxia‐inducing toxic free radicals/ROS, responsible for cellular oxidative stress.^25^ One of the predominant mechanisms governing iron's association with cancer initiates as a protective mechanism of the cell's intrinsic property to eliminate superoxides (O2^•‐^), identified as the primary ROS molecule generated from essential cellular metabolic processes. However, given the proactive metabolic state of tumour cells, overly accumulated ROS along with overall enhanced oxidative stress promote accumulation of endogenous free radical, hydrogen peroxide (H_2_O_2_), a step catalysed by an antioxidant, superoxide dismutase which in presence of excess Fe^+2^ generates a hydroxyl radical (OH^−^) flux through a process called Fenton reaction.^26^, ^27^ These free radicals, in turn, have the ability to drive lipid peroxidation and loss of membrane integrity, an outcome identified as a common event favouring both pro‐tumorigenesis and anti‐tumorigenesis, the fate being majorly dependent on comparative ROS levels against cellular antioxidant defence (AOD).^26^, ^27^ Given this mode of action as schematically represented in Figure 3, while iron‐generated free radicals may stimulate anti‐tumorigenic signalling and drive oxidative stress‐promoted tumour clearance, cancer cells often have the ability to adapt to the hypoxic environment, triggering cellular transformation pathways facilitating their survival and proliferation.^25^, ^28^

ROS's anti‐tumorigenic effect acting through iron‐induced accumulation of lethal amounts of lipid peroxides as a result of Fenton's reaction can be attributed to ferroptosis, an iron‐dependent non apoptotic cell death also identified as an outcome of unrestricted oxidative stress via erastin‐induced inactivation of an antioxidant enzyme, glutathione peroxidase 4 (GPX4).^29^ However, while ferroptosis appears to be a favourable outcome in terms of cancer treatment, it has the ability to induce significant damage to healthy cells while promoting degenerative pathological defects, including carcinogenesis, owing to the declining cellular ability to repair lipid peroxide damages.^30^, ^31^ Furthermore, ferroptosis in cancer cells have been reported to induce prostaglandin E2 release thus affecting its role in immunosuppression, in turn driving chemotherapy resistance and tumour cell proliferation.^32^ Besides, the interplay of the intrinsic antioxidant machinery of cancer cells and administration of iron chelators as an initial part of chemotherapy may synergistically act to hinder or neutralise the positive impact or induction of ferroptosis.^30^ On the other hand, with antioxidant defence used as a common death evading strategy by cancer cells to regulate ROS, contrasting reports on association of reduced production of the antioxidant enzyme, catalase has also been linked to iron deficiency‐related ROS elevation, both in vivo and in vitro, thus fabricating a reconstructed pro‐tumour redox balance, promoting their invasion and therapy resistance.^33^, ^34^ This has again been paradoxically attributed to ROS‐induced lipid peroxidation that causes DNA adducts driving loss of cell membrane integrity and has ultimate serious implications in cancer progression.^21^

Emanating from an imbalanced pro‐oxidant and antioxidant setting, oxidative stress, therefore remains one of the critical components in states of iron deficiency and overload, thus extending its effects on several pathophysiological and chronic complications, including malignancy.^4^, ^35^, ^36^ As one of the major drivers of iron deficiency, oxidative stress principally targets erythrocytes due to their oxygen‐carrying ability, enforcing immediate erythrocyte loss through eryptosis.^35^ Eryptosis along with increased oxidative damage through higher phospholipid externalisation was reported in patients co‐affected with type II diabetes mellitus and chronic kidney disease (CKD), highly predisposed to developing liver, pancreatic, bladder, breast and colon cancers.^37^, ^38^, ^39^ Furthermore, reports of increased eryptosis have been outlined in lung cancer, myelodysplastic syndrome and intestinal tumours in mice.^40^, ^41^, ^42^ However, iron deficiency might not be the sole contributor to oxidative stress.^43^ For example, under conditions of chronic inflammation, iron absorption gets significantly reduced as a defence strategy for iron deprivation to iron‐feeding microbes to control their survival through impairment of ROS detoxification.^6^ Irrespective of the cause, symptomatic anaemia is treated as simply a state of iron deficiency and clinically counteracted by dietary, parenteral and/or enteral iron supplementation and blood transfusions, to restore iron stores for erythropoiesis.^44^

Iron supplementation strategies, however, require the knowledge of underlying causes of iron deficiency in patients, through a series of iron determination tests listed under Table 1, to evade negative effects of iron build up, which might otherwise increase iron overload‐associated risks.^11^, ^45^, ^46^, ^47^, ^48^, ^49^, ^50^, ^51^, ^52^, ^53^, ^54^, ^55^, ^56^, ^57^, ^58^, ^59^, ^60^, ^61^, ^62^, ^63^, ^64^, ^65^, ^66^ There have been few reports highlighting risks of iron accumulation resulting from excess iron ingestion as a part of diet or supplements.^67^, ^68^, ^69^ By 1989, physicians/doctors reported excessive iron as a potent risk associated with cancer, at levels significantly lower than estimated safe in the past.^70^, ^71^ This was later supported by nutritionists recommending limiting consumption of iron‐rich fortified foods, to avoid its over‐absorption in the body.^72^, ^73^ Evidences of clinical iron overload in adults taking daily iron supplements for 7, 15, 35 and 61 years showed a heterogeneous *HFE*‐based phenotypical and genotypical association.^69^ Another study reported ferritin elevation in a postmenopausal Caucasian female, pre‐treated for breast cancer, supplemented with a daily oral dose of 325–975 mg ferrous sulphate for three decades, despite no records of *HFE* mutation and chronic liver disease.^68^ Diet‐induced iron overload risks have also been confirmed through epidemiological and meta‐analytical evidence reporting positive correlation between excess red and processed meat ingestion and increased risks of gastric, colorectal, prostate, lung, breast cancers and renal cell carcinoma, including other chronic disorders like coronary heart failure in patients with type II diabetes.^74^, ^75^, ^76^, ^77^, ^78^, ^79^, ^80^ Higher red meat intake in premenopausal women has also been observed to increase progesterone and oestrogen receptor positive breast cancer risks.^81^ Moreover, excessive red meat consumption (seven times per week) in subjects with elevated Tf levels has been associated with higher mortality.^82^ Red meat carcinogenicity has been attributed to its heme iron content, which due to its structural composition, allows iron to escape absorption inhibition, thus leading it to contribute to >10% of the absorbed iron.^83^

**TABLE 1:** Diagnostic markers' status as reviewed for clinical iron deficiency in anaemic patients ^11^, ^45^, ^46^, ^47^, ^48^, ^49^, ^50^, ^51^, ^52^, ^53^, ^54^, ^55^, ^56^, ^57^, ^58^, ^59^, ^60^, ^61^, ^62^, ^63^, ^64^, ^65^, ^66^

Diagnostic variables	Significance in body iron detection	Advantages	Limitations
Serum ferritin	Iron storage marker and an acute phase reactant. Measures stored iron in liver and spleen.	Inversely correlates with iron assimilation in erythrocytes, cut‐offs for which, vary considerably among experts. Limited evidence suggests that a serum ferritin > and < than 15 μg/L marks high and low iron stores, respectively in patients aged over 5 years.	Sensitive only until body iron stores are depleted and is significantly influenced by infection and inflammation. May be independent of changes in iron stores, thus challenging the interpretation of infection‐driven body iron index. False normal levels may appear in cancer and other chronic conditions.
Soluble transferrin receptor (sTfR)	Regulates Fe‐Tf uptake by erythrocytes. Measures biologically available iron.	Marks the magnitude of erythropoiesis and iron requirement in the body. sTfR levels increase with depletion of iron reserves in IDA, indicating severe iron deficit, therefore, applicable for FID estimation, post iron store depletion.	Remains a critical erythropoietic determinant. sTfR‐logarithmic ferritin ratio opted as a promising index to determine changes in both iron stores and functional iron. The sTfR‐ferritin determination, however, includes high cost and lack of normalisation.
Transferrin saturation (TSAT)	TSAT < and > 20% indicate iron deficiency and overload, respectively. Measures the number of iron‐binding sites on Tf‐Fe.	May be applied to uninflamed patients to identify a normal iron supply to tissues and bone marrow.	Remains an older diagnostic tool, due to its sensitivity to inflammation.
Hepcidin	Hepatic peptide which acts by binding to FPN. High plasma hepcidin indicates iron‐restricted anaemia, including anaemia associated with inflammation, CKD and metastasis in cancers. Deficient hepcidin leads to iron overload conditions in HH, and inefficient erythropoiesis. Systemic hepcidin production is transcriptionally coordinated by plasma iron content, leading to its overproduction in conditions of iron abundance.	Preferred in clinical applications for erythrocytic iron assimilation.	Greatly affected by a range of stimuli, such as hypoxia and/or inflammation, thus making it a dynamic component.
Total iron‐binding capacity (TIBC)	Measures serum Tf (apo, mono and diferric) responsible for iron transport to RBCs or body stores.	Tool in immunological assays used for iron status determination.	May appear as false deficient in conditions of coexisting acute and chronic infections. False normal levels may appear due to hypoproteinaemia.
Gamma‐glutamyl transpeptidase/ gamma‐glutamyltransferase (GGT)	A liver enzyme involved in glutathione metabolism and transport of amino acids and peptides. Correlates with iron toxicity, disease risk and mortality.	Early predictive marker for a multitude of chronic diseases.	Significantly altered in a wide range of diseases and dependent on several iron markers, hence cannot be used as a single‐point measurement.
Complete blood count (CBC)	Measures RBC, white blood cells (WBC), platelet count, Hb and haematocrit (Hct). Also includes mean corpuscular volume (MCV) as a characteristic to RBC size, mean corpuscular Hb concentration (MCHC), indicating Hb amount per RBC.	Evaluates overall health and underlying diseases. MCV determines the underlying aetiology of specific anaemia type.	Individually unable to assess acute changes in iron availability, secondary to ESA therapy. Iron replenishment from RBC pool gets significantly compromised causing suboptimal iron supply to erythroid marrow due to iron store depletion. Inefficient iron supply causes impairment of Hb production, thus leading to low MCHC detectable post weeks of Hb decline.
Serum erythropoietin concentration	Principal regulator of RBC production. Correlates with body iron, serum unsaturated iron‐ binding capacity and ferritin in haemodialysis patients	Identifies the underlying causes of blood conditions.	Iron or vitamin deficiencies in patients may interfere with results.
Percentage of hypochromic red cells (% HRC)	Provides an accurate determination of FID and latent iron deficiency (LID) through the Hb production by RBCs and reticulocytes.	A long‐life span of mature circulating RBCs allows iron status of RBCs. Iron‐restricted erythropoiesis can be determined by %HRC in ACD patients.	Analysis must be done in a fresh sample to avoid errors resulting from RBC swelling.
Reticulocyte haemoglobin content (CHr)	Predicts FID in patients undergoing ESA therapy, determining response to intravenous iron administration in CKD patients, independent of acute phase.	Cost‐effective diagnostic factor for early detection of iron availability for erythropoiesis alongside quality assessment of reticulocytes.	Lacks consistency in cut‐off values determining outcomes in patients, therefore requiring further investigation.
RBC protoporphyrin	Measures compromised iron supply in peripheral RBCs.	Reflects iron supply during RBC production, derived through its accumulation in RBC precursors as a response to low iron.	Accumulation of its levels are also recorded for lead poisoning, hence requires exclusion to lead exposure. Its levels are also raised only several weeks post low‐iron erythropoiesis.
Zinc protoporphyrin	Stable screening marker for iron deficiency measured through systemic iron supply to bone marrow erythrocytes.	Instant, sensitive and cost‐effective measurement and can be used in combination with haemoglobin concentrations for assessment of iron status in population‐based studies.	Its evaluation in iron‐deficiency associated with certain chronic diseases such as uraemia is mainly derived from cross‐sectional studies, and hence, may produce conflicting results, therefore failing to validate its significance as a determinant in such chronic conditions.
Bone marrow aspiration with iron staining	Evaluative measure for elimination of condition of IDA rather than a diagnostic factor.	Used as a confirmatory test in conditions where low TSAT and ferritin levels are at borderline levels associated with disorders other than IDA	Invasive, and decreased predictive accuracy from bone marrow samples with low or unavailable iron in bone marrow samples.

Abbreviations: ACD, Anaemia of chronic diseases; CKD, chronic kidney disease; CBC, complete blood count; ESA, erythropoiesis‐stimulating agent; FPN, ferroportin; FID, functional iron deficiency; GGT, gamma‐glutamyl transpeptidase/gamma‐glutamyltransferase; Ht, haematocrit; HH, hereditary hemochromatosis; IDA, iron deficiency anaemia; LID, latent iron deficiency; MCHC, mean corpuscular Hb concentration; MCV, mean corpuscular volume; % HRC, percentage of hypochromic red cells; RBCs, red blood cells; CHr, reticulocyte haemoglobin content; sTfR, soluble transferrin receptor; TIBC, total iron‐binding capacity; Tf, transferrin; TSAT, transferrin saturation; WBC, white blood cells.

Resulting iron overload has been reported to increase ROS levels, imposing inhibitory effects on haematopoietic cells in iron‐overloaded mice models, via NOX4/ROS/P38 MAPK signalling cascade, a prominent pathway involved in cancer chemokinesis.^84^, ^85^ Additionally, increased oxidative stress as a response to hypoxia in a growing tumour, is a prominent hallmark in solid tumours and is negatively correlated to cancer prognosis and patient survival, the association being directly attributed to hypoxia‐regulated downstream signalling molecules, mainly hypoxia inducible factor‐2 (HIF‐2), responsible for cell cycle arrest and therapy resistance.^86^, ^87^, ^88^ Oxidative stress, therefore, acts as a prominent dual way driver in response to states of iron deficiency as well as overload, through a multi‐directional manner, sometimes acting simultaneously in promoting tumorigenesis. As an evidence, a co‐action of iron deficiency and overload through menstruation‐driven iron loss and menopause‐driven iron accumulation was observed in pre‐ and postmenopausal women, respectively, suffering from breast cancer.^3^ The study inferred that while iron deficiency significantly induced the ROS‐regulated angiogenic molecule, vascular endothelial growth factor (VEGF), iron overload was responsible for ROS accumulation and mitogen‐activated protein kinase (MAPK) activation, thus playing a simultaneous role in breast cancer development and recurrence.^3^

Taken together, the conflicting role of iron‐driven ROS acts synergistically, posing a challenge in cancer therapeutic success, owing to the undefined intersections of states of iron deficiency and overload. While antioxidant therapies aimed towards ROS downregulation are available, they have been shown to enhance metabolic syndromes, along with increased cancer risks and patient mortality.^89^ These outcomes might be attributed to the antioxidant conditions being exploited by cancer stem cells (CSCs) to survive in a tumour microenvironment through minimising DNA damage and promoting metastasis.^90^ This mechanism is chemotherapeutically targeted through increasing ROS and inhibiting the antioxidant machinery in cancer cells.^91^ However, reports on ROS‐independent chemotherapy have questioned the facet of ROS being a direct effector of cell death or merely an outcome of chemotherapy‐induced tumour cell death.^92^ Nonetheless, chemotherapy‐induced ROS drives genetic instabilities in cancer cells, eventually contributing to an evolved heterogenic population of drug resistant and highly metastatic tumour cells.

It is, therefore, crucial to diagnose the underlying factors of iron disbalance, which is often disregarded due to indistinguishable external symptoms, thus demanding independent discussion on the engagement of iron deficiency and overload in cancer.^93^

## 4 IRON DEFICIENCY IN CANCER: CANCER‐RELATED ANAEMIA

Anaemia as defined by the World Health Organisation (WHO) is a ‘*a condition represented by inadequate quantity of RBCs or their oxygen‐carrying ability to suffice the physiological requirements of the body*’.^94^ Iron deficiency anaemia (IDA), one of the most established types of anaemia is characterised by iron levels less than required for normal physiological functions, with symptoms including fatigue, appetite loss, dyspnea, pale skin, shortness of breath and headache.^95^ According to a global survey in 2010, one‐third of the population was affected by anaemia with approximately 50% of the cases stemming from iron deficiency.^96^ The diagnostic cut‐off criteria for IDA in terms of Hb levels, recommended by WHO, ranges from >10–11.5 g/dl to > 12.5–13.8 g/dl in females and males respectively.^97^ IDA's complex pathogenesis might be driven by a lowered erythropoietin response, decreased RBC survival along with reduced iron absorption and macrophageal iron retention, limiting iron delivery to erythroid precursor cells.^98^

Anaemia and cancer, although, being separate health conditions have often been linked, due to the ability of cancer cells to sequester iron, thereby limiting iron availability for RBC production.^15^, ^99^ Anaemia is characteristically prevalent in cancer patients, of practically all cancer types to varying grades, resulting in low quality of life and poor prognosis.^15^ A single‐centre study on patients with solid tumours and hematologic cancers revealed iron deficiency and anaemia in 42.6% and 33% of the subjects, respectively, with highest iron deficiency rates in pancreatic, colorectal and lung cancers.^100^ Cancer‐related anaemia can be a direct consequence of cancer invasion, intrinsic or iatrogenic blood loss, radiation or chemotherapy, bone marrow failure, necrosis or inflammation.^5^, ^98^ Besides, secondary or myelophthisic anaemia may result from cancer invasion‐driven blood loss, erythropoiesis inhibition through tumour marrow infiltration (TMI), or inflammation leading to FID.^5^, ^101^

TMI is responsible for cancer relapse post‐treatment, attributed to dormant cancer cell population leading to minimal residual disease, predicted to be driven by migration of disseminated cancer cells to bone marrow, thus posing challenges to the clinical cancer diagnosis.^102^ TMI‐driven tumour recurrence was corroborated in a recent investigation indicating a rapid cancer relapse with brain metastatic lesion in a patient originally diagnosed with stage III rectal cancer.^103^ Incidentally, another equivalent criterion in cancer‐associated anaemia stems from CIA, comprising a combined action of TMI, blood loss and erythropoiesis inhibition, synergistically co‐driving normal cells into a state of malignancy, as a reaction to inflammation.^5^ Evidence on the higher incidence and relative risks (RR) of anaemia induced by appropriate cornerstone chemotherapeutics have been reported in lung and pancreatic cancer patients subjected to tyrosine kinase inhibitors namely erlotinib and gefitinib.^104^ Besides, low‐ and high‐grade anaemia incidence in breast cancer patients, ranging between 1% and 80%, has also been associated with mainstream combination chemotherapy including 5‐fluorouracil, doxorubicin, etoposide, mitoxantrone, methotrexate and cyclophosphamide.^105^ While iron deficiency has also been reportedly associated to the development or recurrence of certain types of malignancies, such as gastrointestinal carcinomas, lung, urinogenital and premenopausal breast cancer, mild to acute CIA (Hb level < 10 g/dl) has been reported in patients affected with solid tumours in breast, colon/rectum, lung, ovary and stomach.^106^, ^107^, ^108^ Another study with a set of cancer patients undergoing chemotherapy reported chemotherapy‐driven iron loss in 75% of the subjects, with 60% of them diagnosed with AID monitored through low ferritin.^109^ CIA, however, is dependent on tumour grade, type and patient's heart and lung conditions and their exposure to prior anticancer therapies, thus favouring personalised treatments accompanied by management of nutritional deficiencies.^110^ These reports were validated in a study on a range of cancer victims receiving chemotherapy, in which grade 2+ (Hb < 10 g/dl) CIA incidence ranged from about 26% to 59% in colorectal and ovarian cancer, respectively, whereas severe anaemia (Hb <8 g/dl) was maximum in ovarian cancer (about 17%) and minimum in colorectal cancer (4%).^106^ Additionally, there was an overall increase of about 1.7‐fold in grade 2+ CIA risk in stage I to IV transition.^106^

Furthermore, chemotherapy‐induced IDA is a common contributor to renal injury or CKD, resulting through tumour invasion or as an age‐driven atrophy in elderly cancer patients.^5^, ^111^ IDA in CKD or end‐stage renal disorder (ESRD) patients undergoing haemodialysis, develops as a result of imbalanced intestinal iron absorption, chronic inflammations, blood loss and iron overutilization for erythropoiesis spiked through erythropoiesis‐stimulating agents (ESA).^112^ Unstable body iron indices in such patients make it challenging to accurately determine the iron status, due to possible coexistent inflammations or infection and conditions of comorbidity.^113^ ESA dosage optimisation through use of intravenous (IV) iron in CKD following haemodialysis, for chronic anaemia management escalated since 1990, owing to improved outcomes, ease of administration and infrequent adverse concerns, leading to its rapid adoption in the standard treatment procedure.^114^ However, since iron regulation is a closed system lacking an active excretory channel and predominantly controlled by hepcidin, chronic IV iron therapies may pose a risk of iatrogenic iron overload, as reported in a chemotherapeutically treated 56‐year‐old breast cancer patient diagnosed with ESRD.^115^, ^116^ Despite being clinically asymptomatic to iron overload‐induced organ failure, a significantly high iatrogenic iron level was observed as a response to IV iron supplementation for treating kidney transplantation‐associated FID.^116^ Furthermore, IV iron has been reported to increase free iron pool in circulation or tissues through saturation of iron‐binding sites of ferritin and Tf, thus enhancing oxidative injury, mitochondrial damage and cytotoxicity as observed in vitro, in vivo and clinically.^114^, ^117^, ^118^ A recent investigation revealed a concomitant increase in hepatic iron concentration in ESRD patients undergoing dialysis subjected to IV therapy, otherwise observed to significantly decrease on its withdrawal or considerable dose reduction.^119^ However, while contrasting evidence on IV iron therapies significantly reducing risks of transfusion‐based iron overload are available, therapy‐induced erythropoiesis tends to utilise bioavailable functional body iron, creating an iron‐restricted environment, further worsening FID in haemodialysis patients through constant increasing free iron demands.^15^, ^120^, ^121^ Altogether, the clinical use and long‐term safety of ESA and IV iron therapies remain controversial.^114^, ^115^ Relevant reports revealing haemosiderosis in patients undergoing dialysis resulted in re‐investigation of iron overload risks in CKD patients suffering from ESRD and inflammatory bowel disease (IBD), as a response to iron supplements authorised for marketing.^115^ Furthermore, long‐term safety of these therapies was challenged due to a lack of randomised clinical trials with multiple dosage protocols and insufficient and contradictory answers to their efficacy, confirming that the frequency of accumulative iron exposure may be extensively underestimated in haemodialysis patients.^115^ Collectively, an oversimplification of heterogeneous IV iron treatment course in haemodialysis patients reveal that progressive iron exposures may not orient well with iron administration strategies in clinical practises.

Nonetheless, with anaemia simply stemming from a multitude of factors responsible for hindering oxygen delivery to tissues, it is noteworthy that while anaemia is not a sole indicator of cancer, it might develop as an independent or a combined outcome of the disease itself or its therapy. Anaemia in cancer patients, therefore, might not be a direct outcome of cancer, thus making anaemia a symptom rather than a direct diagnostic factor. Besides, anaemia initiating as a stress erythropoietic event may transition to a state of iron overload through concurrent hepcidin decrease, thus revealing an inevitable link between the two states.^122^ Moreover, conditions of anaemia do not necessarily indicate blood transfusions or iron supplementation, which if unchecked, may cause consequential superfluous risks of iron overload, an argument that has been elaborately discussed below, in terms of chronic infections, including cancer.

## 5 IRON OVERLOAD IN CANCER

Iron overload has been widely associated with chronic diseases or pathologies, such as, liver cirrhosis, hemochromatosis, heart failures, type II diabetes, neurodegenerative diseases and cancer, particularly hepatocellular, colon, colorectal, breast, prostate, pancreatic, bladder and lung cancers.^123^, ^124^ While not much has yet been elucidated on the fundamental effects of iron overload in driving tumorigenesis apart from iron overload‐induced oxidative stress‐driven DNA damage and lipid peroxidation, there exists an elementary explanation to the direct association of iron overload to chronic infections, including cancer. Infection is an inter‐tissue entry of pathogenic microbes in multicellular hosts.^125^ Under healthy conditions, iron is stored in proteins like Tf and lactoferrin as a host defence strategy to restrain its availability to pathogens.^125^ Contrarily, patients with clinical iron overload are susceptible hosts to lethal infection causing microbiota, such as *Escherichia coli*, *Vibrio vulnificus* and *Yersinia enterocolitica*, possessing a tendency of iron sequestration from host body in exchange of rapid growth.^125^ Iron supplementation significantly increases unabsorbed soluble free iron, which may be utilised by potentially pathogenic microbial strains to initiate or aggravate infection.^125^ This was revealed in a set of Ivorian subjects fed with iron‐fortified biscuits resulting in a remarkable increase in pathogenic *Enterobacteria* strain along with a growth retardation in beneficial *Lactobacilli* and *Bifidobacteria* strains.^126^ Moreover, higher levels of calprotectin, a gut inflammatory marker were observed in this group's faeces, validating the link between excess iron and inflammation.^126^ Besides, epidemiological evidence suggests a direct association of high serum iron and ferritin with infections progressing to cancer risks, rendering patients less likely to recover and develop treatment resistance.^127^, ^128^ Infection‐associated chronic iron overload has also been reported to drive secondary hemochromatosis, a potent cancer antecedent, also significantly affected by oral iron intake.^68^ With elemental iron proven to be toxic at an oral dosage of over 60 mg/kg, excess dietary iron has been associated with increased systemic levels of heme and non‐heme iron along with circulating free/ferrous ions in the body.^129^, ^130^, ^131^ The evidence of association of oral iron to cancer risks dates as early as 1990s, reported in prospective animal studies revealing incidence of colorectal, hepatic, renal tubular cell carcinomas and sarcomas.^132^, ^133^, ^134^ While related experimental evidence on human subjects were earlier deemed unethical, genetic defects on oral iron assimilation have been proved to be a source of hepatic and non‐hepatic iron overload in certain populations (Whites and sub‐Saharan Africans), as a result of hereditary hemochromatosis.^135^, ^136^, ^137^ However, while hemochromatosis in white populations (North Europeans, Australians predominately Caucasians and French), with no evidence of inbreeding is generally inherited through *HFE* gene in *HLA* locus, iron overload conditions in sub‐Saharan African population has been believed to result solely due to increased intake of traditional home‐brewed beer, rich in non‐heme iron.^138^, ^139^, ^140^, ^141^ Moreover, emerging evidence on hemochromatosis patients has indicated ferritin's role in states of severe shock, causing its rapid release into circulation thus establishing a possible link between iron overload shocks and resulting infections.^142^, ^143^ Additionally, hemochromatosis‐driven iron overload induces epigenetic modification as an initiating event in malignancy.^144^ In response, excess labile iron pool significantly stimulates cancer cell metastasis, as demonstrated in breast cancer through heme oxygenase‐1 (HO‐1) expression.^127^, ^145^

Besides, an increased probability of iron overload risks has been recently established, as a repercussion of iron administration in populations, where iron deficiency was not a causative factor of anaemia.^146^ An ongoing study targeting childbearing‐aged Combodian women highly susceptible to inherent and iron supplementation‐driven iron overload risks due to genetic Hb disorders aims to distinctively determine the effects of common and newer iron supplements, namely ferrous sulphate and ferrous bisglycinate, respectively on ferritin levels and inflammation.^146^ While the study results are pending, its objective remains highly significant to validate the safety and efficacy of iron supplements, as successfully revealed by an earlier study showing improved haematological profile, decreased inflammation and unaffected oxidative stress in anaemic pregnant females supplemented with 120 mg/day of elemental iron as opposed to higher oxidative stress and inflammation in non‐anaemic pregnant females exposed to half the dose.^147^ While serum ferritin remained unaltered in the non‐anaemic category, the study reflected upon iron overload toxicity as a possible cause of elevated radical generation and inflammation identified as potent initiators of malignancy.^147^

Elevated free radical generation as a response to iron overload promotes cancer cell survival and metastasis by causing genetic aberrations and mitochondrial dysfunction acting in tandem or independently.^148^ Therapy resistance, one of the principal strategies of cancer cell survival has been associated with ROS‐driven dysfunction in mitochondrial morphology and downregulation of the mitochondrial enzyme, pyruvate dehydrogenase, along with epithelial to mesenchymal transition (EMT), reported in gefitinib‐resistant lung adenocarcinoma H1650 clones.^148^ Moreover, iron‐induced ROS‐driven overexpression of a reported metastatic and prognostic cancer marker, caveolin‐1, known to promote resistance to chemotherapy‐induced apoptosis, was reported in H460 lung carcinoma, on exposure to ferrous sulphate.^149^ Furthermore, with CSCs being considered as the primary sub‐population responsible for cancer relapse, metastasis and therapy resistance, recent investigations have reported the role of iron not only in promoting tumorigenesis, but also stemness in tumour cells, as has been validated by an elevated TfR1 expression in breast CSCs as compared to differentiated cancer cells.^127^, ^150^ The survivability of CSCs is attributed to the presence of an integral antioxidant machinery, CD44 marker variant, to help them maintain low levels of intracellular ROS to evade their toxic effects.^150^ Moreover, the involvement of CD44, being a promoter of EMT, indicates the role of iron in the tumorigenesis transition.^127^, ^151^ EMT is responsible for loss of cell adhesion and polarity through downregulation of epithelial marker, E‐cadherin, while upregulating certain mesenchymal and motility markers such as Snail, TWIST, N‐cadherin and vimentin.^152^ Iron deprivation has been shown to reverse the EMT process, as validated by significant downregulation of E‐cadherin in KM12C colon cancer cell as a response to an iron‐regulatory protein, neutrophil gelatinase‐associated lipocalin (NGAL)/lipocalin2.^153^ Moreover, while excess iron in the form of ferric chloride was able to upregulate ferritin levels in NGAL overexpressing KM12C cells, deferoxamine (DFO), an established iron chelator, was able to significantly reduce the ferritin concentration to undetectable limits. These factors collectively portray iron overload as either a causative factor or an outcome of malignancy, irrespective of its source, including unsought iron administrations, iron‐rich diet, history of chronic diseases or genetic imbalances in iron‐regulatory system.^153^

Taken together, although the speculation of iron's role in cancer is a labyrinth of several factors, the interdependence of iron deficiency and overload in a heterogeneous tumour environment cannot be disregarded. While not much validation is available on the iron‐restoration therapies driving conditions of iron overload in cancer patients, interestingly, appearing as a symbiotic relationship, relevance of judicious monitoring of iron markers in anaemic patients, prior to opting for iron reinstating strategies, must be emphasised. One of the major requisites to evade undesirable iron accumulation as a response to administered iron is, therefore, to critically identify the possible intersections of both states of iron deficiency and overload, one of the key hindrances to successful therapies in iron‐associated cancer.

## 6 TARGETING IRON METABOLISM FOR CANCER THERAPY

As mentioned earlier in this review, with iron deficiency and overload interplaying conjointly in a cancer patient at varied points, it appears to generate a rather puzzling condition hindering the identification of the exact target for cancer therapy without jeopardising a patient's health. A detailed understanding of the patient's underlying status of iron is, therefore, crucial in order to create a personalised chemotherapeutic regimen.

For instance, if AID has been identified as a potential cause for iron deficiency in a cancer patient, a favourable therapy regimen must include newer or combination therapeutics maintaining lower anaemia incidence and risk rates. One such example has been reported in a meta‐analytical study involving solid tumour patients exposed to appropriate chemotherapeutics alone and in combination with a humanised monoclonal antibody (bevacizumab), with bevacizumab able to significantly reduce both incidence and risk rates of anaemia in a dose‐dependent manner, as compared to chemotherapy alone.^154^ However, with chemotherapy‐related anaemia being a frequent symptom in cancer patients and lack of extensive research on the pathogenetic mechanisms of anaemia development as a response to chemotherapeutics, information on antianaemic therapies remains limited.^104^ Particularly, chemotherapy‐induced lower grade anaemia appears to be more frequent than higher grade anaemia, usually requiring a blood transfusion.^104^ With no approved treatments available for correction of mild anaemia, the only options available for iron restoration remain chemotherapy interruption, iron administration and blood transfusion. However, long‐term iron administrations and blood transfusions may lead to iron deposition in organs eventually enabling iron accumulation in circulation to toxic levels potentially causing a risk of chronic infection, including carcinogenesis.^155^ Recombinant human erythropoietin namely epoetins in combination with chemotherapeutics have been approved as alternatives for correction of anaemia in patients harbouring non‐myeloid tumours, in order to reduce the number of blood transfusions in a cancer patient.^104^, ^105^ Regardless, the risks of iron overload as a response to iron restoration remains a concern in patients merely affected by FID. While use of iron chelators alone and in combination have been widely recognised to check unwanted iron overload in cancer patients, with their role attributed to enhance miRNA precursor processing and upregulate expression and function of tumour suppressor proteins, some of the chelators, namely deferiprone holds a redox potential capable of generating intracellular ROS.^127^, ^156^ Although this might be a favourable step towards driving ferroptosis acting against cancer cells, there have been limited evidence on the success of iron chelators on solid tumours, the reason accredited to their response towards stabilisation and enhanced expression of hypoxia inducible factor‐1 alpha (HIF‐1α), further aiding tumour proliferation, metastasis and angiogenesis.^127^, ^157^ An effective strategy for treatment of solid tumours, therefore, indicates a possible addition of HIF‐1α inhibitor to iron chelation therapy, as reported in one of the studies exploiting a combination of DFO and HIF‐1α inhibitor (lificiguat/YC1) exhibiting a synergistic antitumour effect on pancreatic tumours in vitro and in xenograft mice models.^158^

Nonetheless, it is noteworthy that cancer is a genetic disorder displaying multiple identified and unidentified abnormalities in cellular metabolism. While dysregulated iron metabolism is one of the features in cancer cells driving heightened influx and decreased efflux of iron from tumour cells, it is unclear if this imbalance precedes the cancerous transformation or is a consequential response to cancer, aiding as an adaptive step for maintenance and progression of malignancy. With multiple iron‐dependent paradoxes simultaneously playing differential role in different cancer types and patients, and existing limitations and concerns for each iron modulation approach, it is confusing whether the best possible approach for its therapy lies in inhibition of iron usage or to enhance iron flooding to induce ferroptosis, thus rendering it challenging to identify which patients are most likely to respond to a given therapy. Furthermore, development of novel iron‐based therapeutics remains rigorous owing to their toxicity, rapid metabolism, short half‐lives and increasing resistance of cancer cells.

## 7 CONCLUSION

While iron and ROS are key factors in cellular homeostasis, their disrupted regulation act as a driver in tumorigenesis. Stemming from the dual role of iron in cancer, both states of iron deficiency and overload play a major role in the disease itself, as well as present themselves as an unavoidable treatment‐associated risk. The complexity to decipher the exact functioning of iron as a pro‐cancer or anticancer factor synergistically acts with the puzzling states of iron deficiency and overload in chronic complications, including cancer. Moreover, for majority of physicians, the primary concern in terms of ‘iron’ till date, remains anaemia or simply a state of iron deficiency, without comprehending ‘iron overload’ as a more prevalent and leading issue, and substantially more life‐threatening, leading to a myriad of complications. Although a frequent symptom, pathogenesis of iron deficiency, yet remains poorly addressed in patients with cancer and other chronic infections along with individuals genetically predisposed to iron overload. Unmethodical red cell transfusions and iron supplementations established as default treatment strategies for iron deficiency may open a ‘Pandora's box’, promoting unwanted risks of iron overload, a precursor to tumorigenesis and a condition that is often overlooked. Hence, a detailed understanding of the iron status in patients prior to anticancer or iron‐restoration therapies is a critical requirement to evade exacerbation of a pre‐existing condition. Moving forward, an individualised treatment of iron deficient patients might be preferably opted for, which requires a detailed diagnostic evaluation of iron deficiency and its origin by routine blood testing prior to iron supplementation.

## CONFLICT OF INTEREST

The authors declare no conflict of interest.

## Author contribution

Tulika Basak has contributed to the review article's conceptualisation, literature search, draft preparation, review and editing. Rupinder Kaur Kanwar has contributed to the review article's conceptualisation, literature search, draft revisions and editing. Both authors have read and agreed to the published version of the review article.

## Ethical approval statement

This is a review article based on published literature; no human/animal participation or cell‐based work involved. Ethical statement is not applicable.

## Documentation of informed consent/clinical trial registration/review boards

Not applicable for this review article.

## Data Availability

Data sharing is not applicable to this review article as no new data were created or analysed. The article, however, contains a table and three schematic figures, which are original and have been created by the authors through reviewing the information, pooled from the already published literature/ journal articles. All the references have been duly cited in this manuscript. No third‐party material (figures or tables) has been included in the current article.
